# HPV-16 E7 Interacts with the Endocytic Machinery via the AP2 Adaptor μ2 Subunit

**DOI:** 10.1128/mbio.02302-22

**Published:** 2022-10-18

**Authors:** Om Basukala, Oscar Trejo-Cerro, Michael P. Myers, David Pim, Paola Massimi, Miranda Thomas, Corrado Guarnaccia, David Owen, Lawrence Banks

**Affiliations:** a International Centre for Genetic Engineering and Biotechnology, Trieste, Italy; b Cambridge Institute for Medical Research, University of Cambridge, Cambridge, United Kingdom; Tufts University School of Medicine; Duke University School of Medicine

**Keywords:** HPV, E7, endocytosis, clathrin, AP2

## Abstract

Human papillomavirus (HPV) E7 plays a major role in HPV-induced malignancy, perturbing cell cycle regulation, and driving cell proliferation. Major targets of cancer-causing HPV E7 proteins are the pRB family of tumor suppressors, which E7 targets for proteasome-mediated degradation and whose interaction is promoted through an acidic patch, downstream of the LXCXE motif in E7, that is subject to phosphorylation by casein kinase II (CKII). In this study we show that HPV-16 E7 targets the AP2-complex, which plays a critical role in cargo recognition in clathrin-mediated endocytosis. Intriguingly, HPV-16 E7 contains a specific amino acid sequence for AP2 recognition, and this overlaps the pRb LXCXE recognition sequence but involves completely different amino acid residues. HPV-16 E7 does this by binding to the AP2-μ2 adaptor protein subunit via residues 25-YEQL-28 within the LXCXE motif. Point mutations at Y25 within 22-LYCYE-26 suggest that the interaction of E7 with AP2-μ2 is independent from pRB binding. In cells, this interaction is modulated by acidic residues downstream of LXCXE, with the binding being facilitated by CKII-phosphorylation of the serines at positions 31 and 32. Finally, we also show that association of HPV-16 E7 with the AP2 adaptor complex can contribute to cellular transformation under low-nutrient conditions, which appears to be mediated, in part, through inhibition of AP2-mediated internalization of epidermal growth factor receptor (EGFR). This indicates that E7 can modulate endocytic transport pathways, with one such component, EGFR, most likely contributing toward the ability of E7 to induce cell transformation and malignancy. These studies define a new and unexpected role for HPV-16 E7 in targeting clathrin-mediated endocytosis.

## INTRODUCTION

Infectious agents cause over 15% of human cancers, and one-third of these are caused by human papillomaviruses (HPVs) (1). A small group of HPVs, classified as high risk, cause cancers of the cervix, head and neck, and anogenital region, among others. Cervical cancer is the second leading cause of cancer death in women aged 15 to 44 and is almost always associated with infection by high-risk HPVs (2). More recently, there has been a significant rise in oropharyngeal cancers associated with HPV infection (3). Currently, effective prophylactic vaccines are available against several of the high-risk HPV types (reviewed in reference 4); however, the difficulties linked to the cost and logistics of these vaccines in developing countries—where most of these infections occur—remain the biggest challenges in fighting the cancers associated with these viruses. In addition, as the vaccine has no therapeutic effect; those already infected are at risk of developing cancers later in life, suggesting the urgent need for, and importance of, therapeutic agents (reviewed in references 5 and 6).

HPV infections are, in general, cleared by the host immune system; however, in some individuals a persistent infection develops, which, together with a perturbed immune clearance, marks the highest risk factor for the development of cancer >10 to 20 years later (7, 8). The major hallmark of HPV-associated cancers is the high and continued expression of the HPV oncoproteins E6 and E7, which contribute to the development of these malignancies by targeting several host cellular processes (reviewed in reference 9). Mainly, the E7 oncoprotein binds the pocket proteins (pRB, p130, and p107), allowing E2F transcription-induced cell cycle reentry in already differentiated keratinocytes, while the HPV E6 oncoprotein, by targeting the tumor suppressor p53 for degradation, circumvents the consequent cellular apoptosis. While both these oncoproteins are known to play significant roles in the development and maintenance of cancer, much remains to be clarified in defining the mechanisms behind their roles in malignant transformation (reviewed in reference 10).

**FIG 1:**
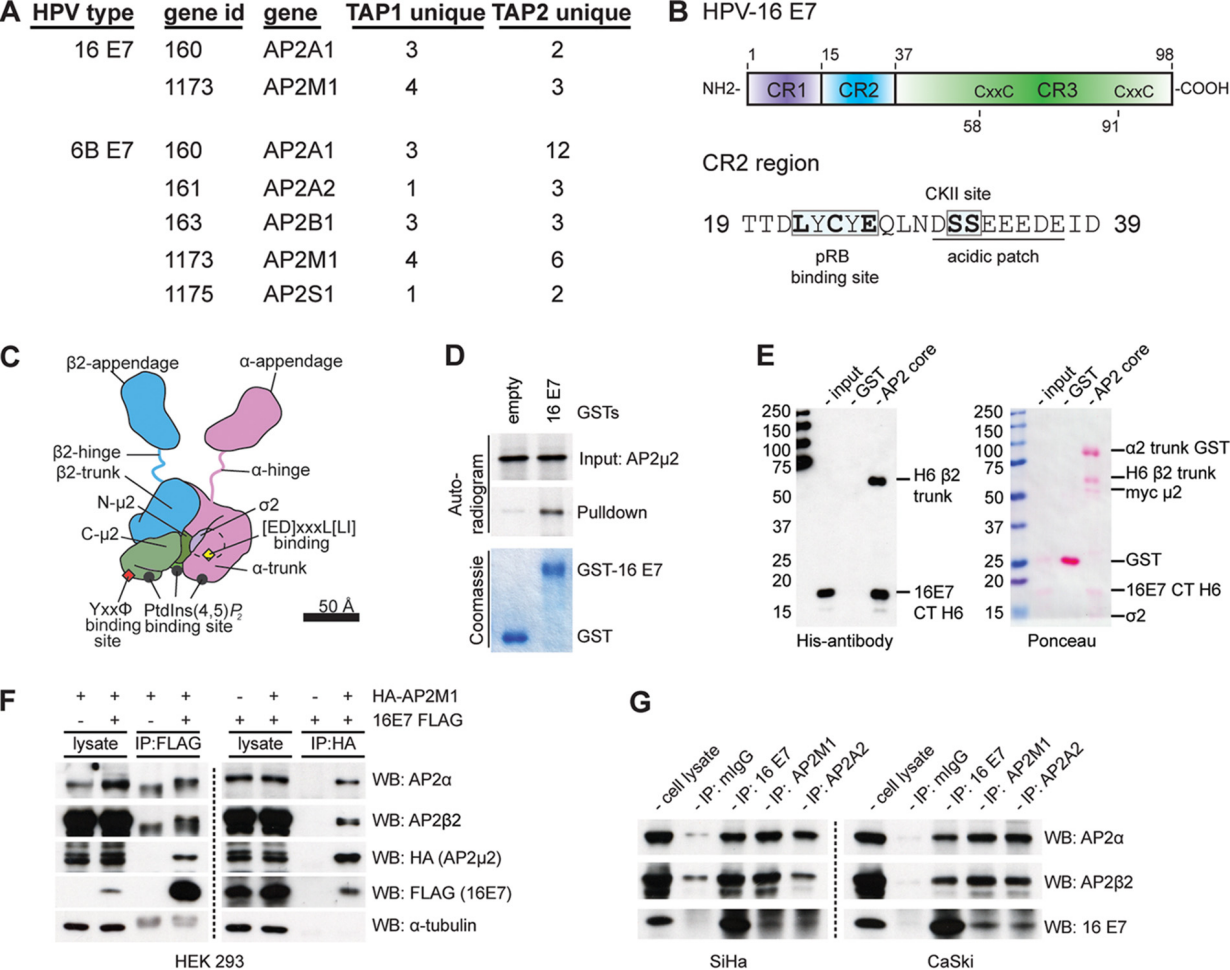
HPV-16 E7 associates with the AP2 adaptor protein complex μ2 subunit. (A) List of adaptor protein subunits showing unique hits in a proteomic analysis of HPV 16 and HPV 6 E7, which was performed by Rozenblatt-Rosen and colleagues and reported in reference 14. (B) Schematic representation of HPV-16 E7 with conserved regions (CR2) shown in detail, indicating the residues responsible for binding with pRB and regions rich in acidic amino acids with serine residues phosphorylated by CKII. (C) Schematic representation of endocytic adaptor protein complex 2 with subunits α, β, μ, and σ, showing regions within them important for membrane binding, cargo binding, phosphorylation site, and clathrin binding. (D) *In vitro* GST pulldown assay. Empty GST and GST-16 E7 agarose beads were incubated with i*n vitro* translated and [S^35^]-radiolabeled AP2-μ2, washed extensively, and then analyzed by SDS-PAGE and Coomassie gel staining and auto-radiogram to observe bound AP2-μ2. (E) *In vitro* binding assay. Purified C-terminally His-tagged HPV-16 E7 was incubated with empty GST or AP2 core GST, washed extensively, and analyzed by SDS-PAGE and Ponceau staining to visualize protein subunits and Western blotting for His-antibody to detect HPV-16 E7. (F) Coimmunoprecipitation assay. HEK 293 cells transfected with C-terminally FLAG-tagged HPV-16 E7 and/or HA-tagged AP2-μ2 were lysed and incubated with anti-HA or anti-FLAG agarose beads as indicated. Purified anti-HA/anti-FLAG agarose beads were analyzed for bound AP2 complex subunits or HPV-16 E7 using SDS-PAGE and Western blotting using the indicated antibodies. (G) Coimmunoprecipitation assay. SiHa and CaSki cell lysates were incubated overnight with mouse anti-HPV-16 E7 antibody or mouse anti-AP2-μ2 or mouse anti-AP2-α antibody, or mouse anti-GFP antibody, and immune complexes were precipitated using protein A agarose beads and extensively washed. Bound complexes were analyzed by SDS-PAGE and Western-blotting for AP2 subunits and HPV-16 E7.

HPV oncoproteins are very plastic in their ability to interact with many host proteins and modulate their functions (reviewed in references 11–13). A proteomic screen (14) using different HPV types showed that HPV E7 interacts with several proteins involved in membrane protein trafficking, suggesting that it could affect the endocytic machinery of the cell (Fig. 1A). Endocytosis is the process of selective packaging and trafficking of membrane proteins into endosomes in the cell interior, a process that is mainly mediated by clathrin and adaptor proteins (AP) (15, 16). Several steps in vesicular trafficking that are important for exchange of proteins and lipids between intracellular membrane compartments (plasma membrane, endosomes, and the trans-Golgi network) use clathrin/clathrin adaptor coats (17). Protein sorting within these membrane compartments mostly relies on short linear peptide sequences (sorting signals) in the cytoplasmic domain of transmembrane proteins, which are recognized by adaptor proteins. These sorting signals include YXXφ (where φ is a hydrophobic residue), [D/E]XXXL[L/I], FXNPXY, DXXLL, and the acidic cluster motif (phosphoserines flanked by acidic residues, often seen adjacent to dileucine motifs). Clathrin adaptor complexes (AP1 to 3) are heterotetramers composed of large subunits (γ1, γ 2/αA, αC/δ and β1/β2/β3A, β3B), a medium subunit (μ1A, μ1B/μ2/μ3A, μ3B), and a small subunit (σ1A, σ1B, σ1C/σ2/σ3A, σ3B) with corresponding gene names designated AP1G1-2/AP2A1-2/AP3D1 and AP1B1/AP2B1/AP3B1-2 for the large subunits, AP1M1-2/AP2M1/AP3M1-2 for the medium subunit, and AP2S1-3/AP2S1/AP3S1-2 for the small subunit. Tyrosine-based motifs bind to the μ subunits, while leucine-based motifs bind to the σ and γ/α subunits (18, 19). The AP2 adaptor complex is mainly involved in the endocytic process, and many cellular proteins contain these endocytic motifs, allowing them to be recognized by the AP2 complex and endocytosed to the endosomes. Intriguingly, a closer look at HPV-16 E7 shows that it contains a YXXφ endocytic motif, partly overlapping the LXCXE motif. This is followed by an acidic cluster motif at the casein kinase II (CKII) phosphorylation site, typical of the classical cytoplasmic tail of furin, which mediates *trans*-Golgi network (TGN) localization and endocytosis at the plasma membrane (20, 21). In this study we therefore asked whether, via this motif, HPV-16 E7 is able to cross talk with the endocytic machinery of the cell.

## RESULTS

### HPV-16 E7 associates with the AP2 adaptor protein complex.

**FIG 2:**
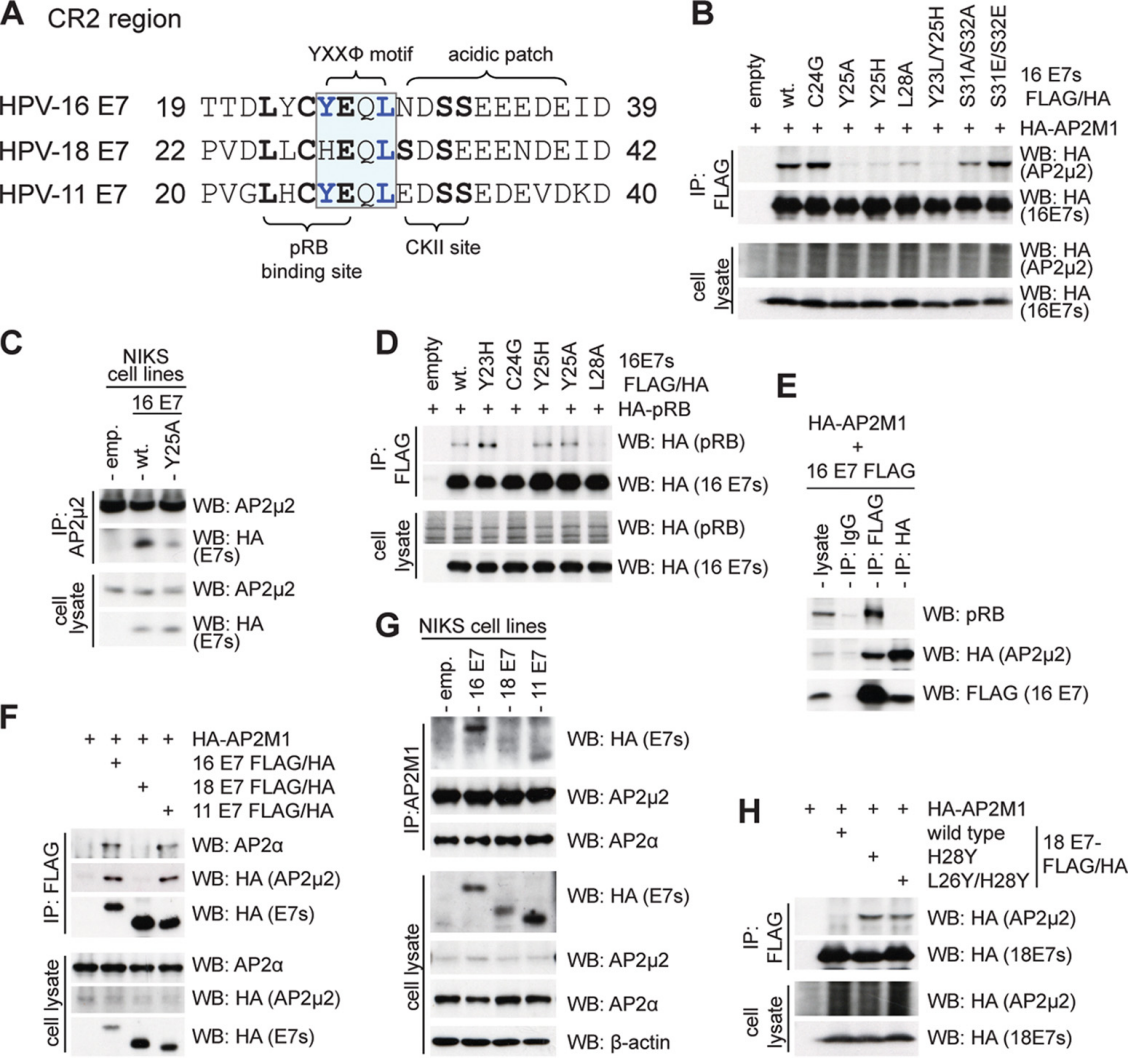
HPV-16 E7 contains a bona fide YxxФ motif interacting with the cargo binding site of the AP2-μ2 subunit. (A) Protein sequence alignment of the CR2 region between HPV-16, -18, and -11 E7s to show the potentially conserved Yxxφ motif partly overlapping with the LXCXE pRB binding site. The location of acidic patch and phosphorylation by CKII is also shown. (B) Coimmunoprecipitation using anti-FLAG agarose beads in HEK 293 cell lysates overexpressing HA-AP2M1 and C-terminally FLAG/HA-tagged HPV-16 wild type and mutants, as indicated, to map residues responsible for binding AP2M1. Immune complexes were analyzed by SDS-PAGE and Western blotting. (C) Coimmunoprecipitation assay showing pulldown of HPV-16 E7s from cell lysates of NIKS-empty, NIKS-16 E7 wild-type, and NIKS-16 E7 Y25A stable cell lines, using mouse anti-AP2-μ2 antibody bound to protein G Sepharose beads and analyzed by SDS-PAGE and Western-blotting for AP2-μ2 (rabbit anti-AP2-μ2 antibody) and HPV-16 E7s (goat anti-HA antibody). (D) Coimmunoprecipitation using anti-FLAG agarose beads and lysates of HEK 293 cells overexpressing HA-pRB and C-terminally FLAG/HA-tagged HPV-16 E7 wild type and mutants, as indicated, to analyze the levels of pRB bound by different mutants of HPV-16 E7. Immune complexes were analyzed by SDS-PAGE and Western blotting. (E) Coimmunoprecipitation assay showing pulldown of pRB using mouse anti-HA or mouse anti-FLAG, or mouse anti-GFP antibody bound to protein G Sepharose beads, incubated with cell lysates from HEK 293 cells cotransfected with C-terminally FLAG-tagged HPV-16 E7 and HA-tagged AP2-μ2. Levels of pRB bound to immune complexes were analyzed using SDS-PAGE and Western-blotting using rabbit anti-pRB antibody; levels of HPV-16 E7 were detected using mouse anti-FLAG antibody, and levels of AP2-μ2 were detected using mouse anti-HA antibody. (F) Coimmunoprecipitation using anti-FLAG agarose beads and lysates of HEK293 cells overexpressing HA-AP2M1 and C-terminally FLAG/HA-tagged HPV-16, -18, or -11 E7, analyzed by SDS-PAGE and Western blotting. (G) Coimmunoprecipitation assay showing pulldown of E7s from cell lysates of NIKS-empty, NIKS-16 E7, NIKS-18 E7, and NIKS-11 E7 stable cell lines, using mouse anti-AP2-μ2 antibody bound to protein G Sepharose beads and analyzed by SDS-PAGE and Western blotting for AP2-μ2 using rabbit anti-AP2-μ2 antibody and HPV-E7s using goat anti-HA antibody. (H) Coimmunoprecipitation using anti-FLAG agarose beads and lysates of HEK 293 cells overexpressing HA-AP2M1 and C-terminally FLAG/HA-tagged HPV-18 wild type and mutants, as indicated, to analyze levels of AP2M1 bound by different mutants of HPV-18 E7. Immune complexes were analyzed by SDS-PAGE and Western blotting.

Early studies of the N-terminal regions of HPV-16 E7 found sequence similarities to some regions of the adenovirus (Ad) E1a protein and the simian vacuolating virus 40 large tumor antigen (SV40T), suggesting that similar mechanisms of action of these viral proteins might be essential for cellular transformation (22). Later, the LXCXE sequence motif in the conserved region 2 (CR2) of HPV-16 E7 was shown to interact with the retinoblastoma tumor suppressor gene product pRB (23), similarly to Ad E1a (24) and SV40T (25), using similar transactivation and transformation functions (22, 26, 27). It is now well established that the CR2 region of HPV-16 E7 is required to modulate many host cell regulatory proteins that are important for the viral life cycle, for cellular transformation, and for maintenance of the transformed phenotype of tumor cells (28–31). Interestingly, the LXCXE sequences in the HPV-16 E7 CR2 (Fig. 1B and 2A) partly overlap the endocytic motif (YXXφ, where X is any amino acid and φ is a hydrophobic residue), which is potentially able to bind to endocytic adaptor proteins. A proteomic screen also identified several of these endocytic adaptor protein complex AP2 subunits (Fig. 1C) as potential binding partners of HPV E7 (Fig. 1A) (14). To further investigate if such an interaction is possible, we did an *in vitro* glutathione *S*-transferase (GST) pulldown assay, in which [35]-S-labeled AP2-μ2 was incubated with purified HPV-16 E7 GST fusion protein, and bound proteins were analyzed by SDS-PAGE and autoradiography. HPV-16 E7 does, indeed, bind to the AP2-μ2 subunit of the adaptor protein complex 2 (AP2) (Fig. 1D). Furthermore, when purified C-terminally, His-tagged HPV-16 E7 was incubated with purified GST AP2 core and analyzed by GST pulldown assay, we could pull down HPV-16 E7, suggesting that there is a direct interaction between HPV-16 E7 and the AP2 adaptor complex, potentially via the AP2-μ2 subunit (Fig. 1E).

We could also see that such an interaction occurs *in vivo*, by overexpressing C-terminally FLAG-tagged HPV-16 E7 and HA-tagged AP2M1 in HEK 293 cells; after 24 h the AP2 adaptor protein complex subunits can be coimmunoprecipitated with FLAG-tagged HPV-16 E7 using anti-FLAG agarose beads and, reciprocally, HPV-16 E7 can be coimmunoprecipitated with AP2-μ2 using anti-HA agarose beads (Fig. 1F). We then extended this analysis to cells endogenously expressing E7 by performing coimmunoprecipitation assays in SiHa and CaSki cells using antibodies against HPV-16 E7, and we showed that the AP2 adaptor protein complex subunits are immunoprecipitated with HPV-16 E7, and a reciprocal immunoprecipitation of HPV-16 E7 is seen using antibodies against AP2-μ2 or AP2α subunits (Fig. 1G), suggesting that HPV-16 E7 is a bona fide interacting partner of the AP2 adaptor protein complex and that it associates potentially via a tyrosine-based endocytic motif in its CR2 region.

### HPV-16 E7 interacts with the AP2-μ2 subunit via 25-YEQL-28 and the CKII phospho-acceptor site at S31/S32.

The HPV-16 E7 residues responsible for binding to the AP2 complex, 25-YEQL-28, appear to partly overlap the LXCXE pRB binding motif (Fig. 2A). To further map this site, several CR2 region mutants of HPV-16 E7 were generated, and these were analyzed by coexpressing them with HA-tagged AP2-μ2 in HEK 293 cells, followed by immunoprecipitation using FLAG agarose beads and Western blotting, as before. We can see from Fig. 2B that the mutations Y25A, Y25H, L28A, and Y23L/Y25H, in the putative YXXφ endocytic motif of the E7 CR2, severely compromise the ability of HPV-16 E7 to bind AP2-μ2, suggesting that these residues are critically important for E7’s interaction with the AP2-μ2 subunit. Furthermore, when a double alanine (S31A/S32A) mutant of HPV-16 E7 is used, there is a decrease in interaction, while a phosphomimic (S31E/S32E) mutant interacts with AP2-μ2 as efficiently as wild-type HPV-16 E7 (Fig. 2B, lane 2 compared with lanes 8 and 9), suggesting that phosphorylation of E7 might play a role in this association. We also validated this interaction using normal immortal keratinocytes (NIKS) stable cell lines expressing either empty vector, HPV-16 E7 wild type, or the HPV-16 E7 Y25A mutant to perform coimmunoprecipitation assays with AP2-μ2 antibody (Fig. 2C). Since these residues partly overlap the LXCXE pRB binding region, we asked if they might also affect the ability of E7 to interact with pRB. Using immunoprecipitation and Western blotting assays, as before, we found that mutating the Y25 residue to histidine or alanine has minimal effects upon pRB binding (Fig. 2D). Furthermore, when we probed for pRB in reciprocal coimmunoprecipitation as before in HEK 293 cells overexpressing HA-AP2M1 and C-terminally FLAG-tagged HPV-16 E7, we could see that pRB immunoprecipitates with E7 but not with AP2-μ2 (Fig. 2E), suggesting that E7’s AP2-binding and pRB-binding activities are separable and, potentially, have evolved to execute diverse biological functions. The L28A mutation, however, seems to severely abrogate the ability of E7 to interact with pRB, suggesting that a conserved Leucine residue beyond the LXCXE motif might have a critical role in interacting with RB (Fig. 2D).

**FIG 3:**
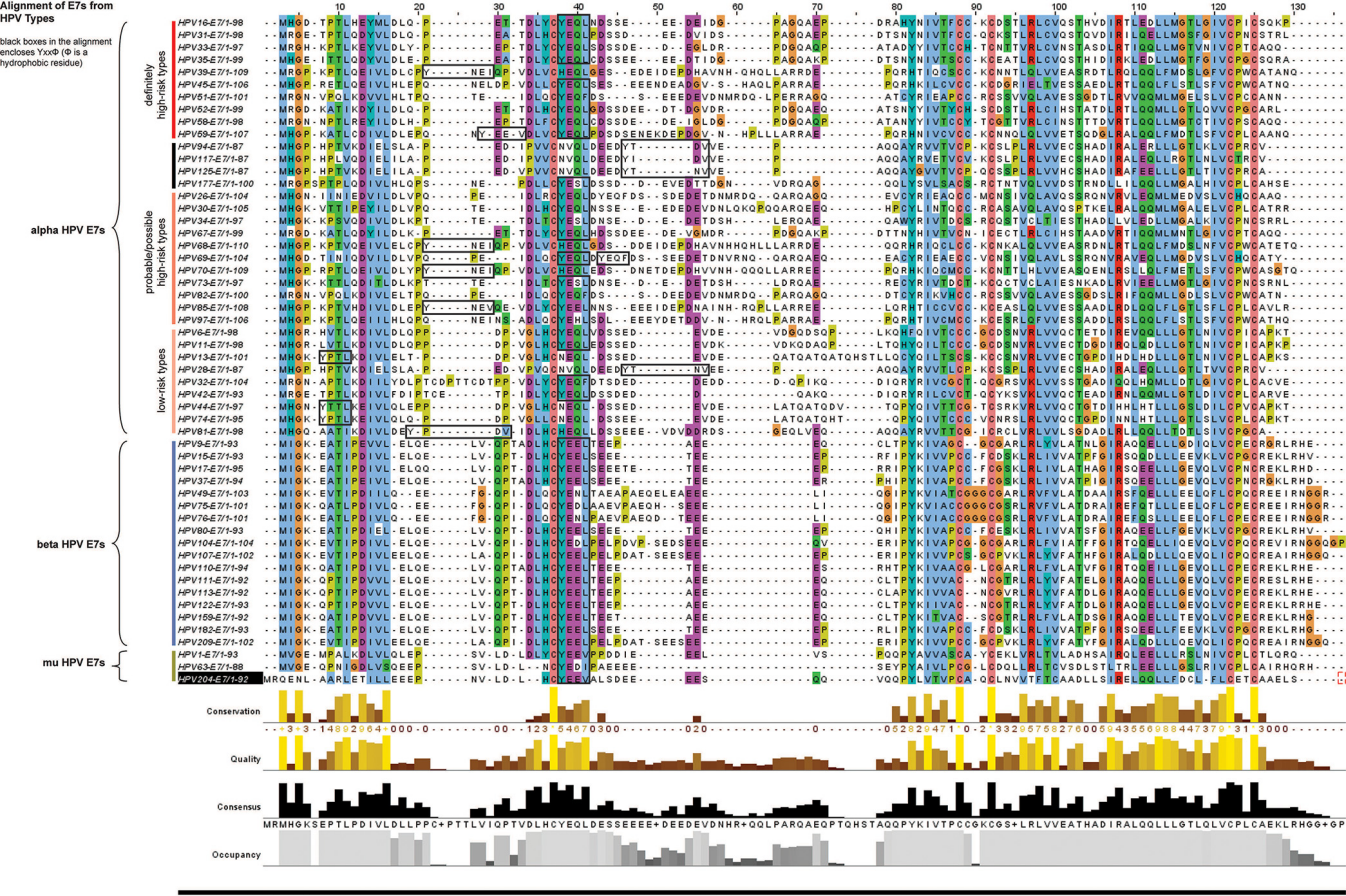
Alignment of E7 proteins from HPV types with a potential AP2-μ2 binding site, showing the YXXΦ motif in the CR2 region (boxed), grouped based on risk types.

We then proceeded to analyze the sequences of multiple HPV-E7s for the presence of an endocytic motif (Yxxφ) in their CR1 or CR2 domains. Interestingly, we found it to be present in the E7 of 54 different HPV types: three Mu HPVs (HPV-1, -63, and -204), seventeen beta HPVs (HPV-9, -15, -17, -37, -49, -75, -76, -80, -104, -107, -110, -111, -113, -122, -159, -182, -209), and thirty-four alpha HPVs, of which ten (HPV-16, -31, -33, -35, -39, -45, -51, -52, -58, and -59) are classified as group 1, cancer-causing high-risk types; eleven (HPV-26 -30, -34, -67, -68, -69, -70, -73, -82, -85, and -97) are group 2, probable/possible high-risk types, and nine (HPV-6, -11, -13, -28, -32, -42, -44, -74, and -81) are group 3, low-risk types (refer to Fig. 3 for an alignment of all 54 motif-containing HPV E7s), which suggests a broadly conserved function for this site in HPV biology.

Interestingly, although most high-risk HPV types have this motif, the HPV-18 E7 has a histidine instead of the consensus Y25 at the putative endocytic motif site (Fig. 2A); we questioned whether this residue would prevent interaction with the AP2 complex, as occurred with HPV-16 E7 when the Y25 residue was mutated (Fig. 2B). We overexpressed the C-terminally FLAG/HA-tagged HPV-16, -18, or -11 E7s and HA-AP2-μ2 in HEK 293 cells and performed a coimmunoprecipitation assay, as before. In Fig. 2F it can be seen that HPV-18 E7 has a dramatically decreased ability to bind AP2-μ2 compared with HPV-16 and HPV-11 E7s. Furthermore, we saw similar differential binding between AP2-μ2 and E7s in the case of NIKS stable lines expressing either empty vector, HPV-16 E7, -18 E7, or -11 E7, using coimmunoprecipitation as before, depending on the presence of the tyrosine residue in the Yxxφ motif (Fig. 2G). We generated further mutants: HPV-18 E7 H28Y and HPV-18 L26Y/H28Y (as an HPV-16 “look-alike” in the LXCXE and Yxxφ regions) to generate a Yxxφ motif, and analyzed their ability to interact with AP2-μ2 using HEK 293 overexpression and coimmunoprecipitation as before. As can be seen in Fig. 2H, swapping a tyrosine residue at 28 for histidine in HPV-18 E7 allows it to interact with AP2-μ2, suggesting that there is a critical requirement for tyrosine at this position, as is true for a typical endocytic motif in cargoes of AP2-μ2 (19, 32).

### Hydrophobic pockets and basic residues in AP2-μ2 are important for interaction with HPV-16-E7 in a phosphorylation-dependent manner.

**FIG 4:**
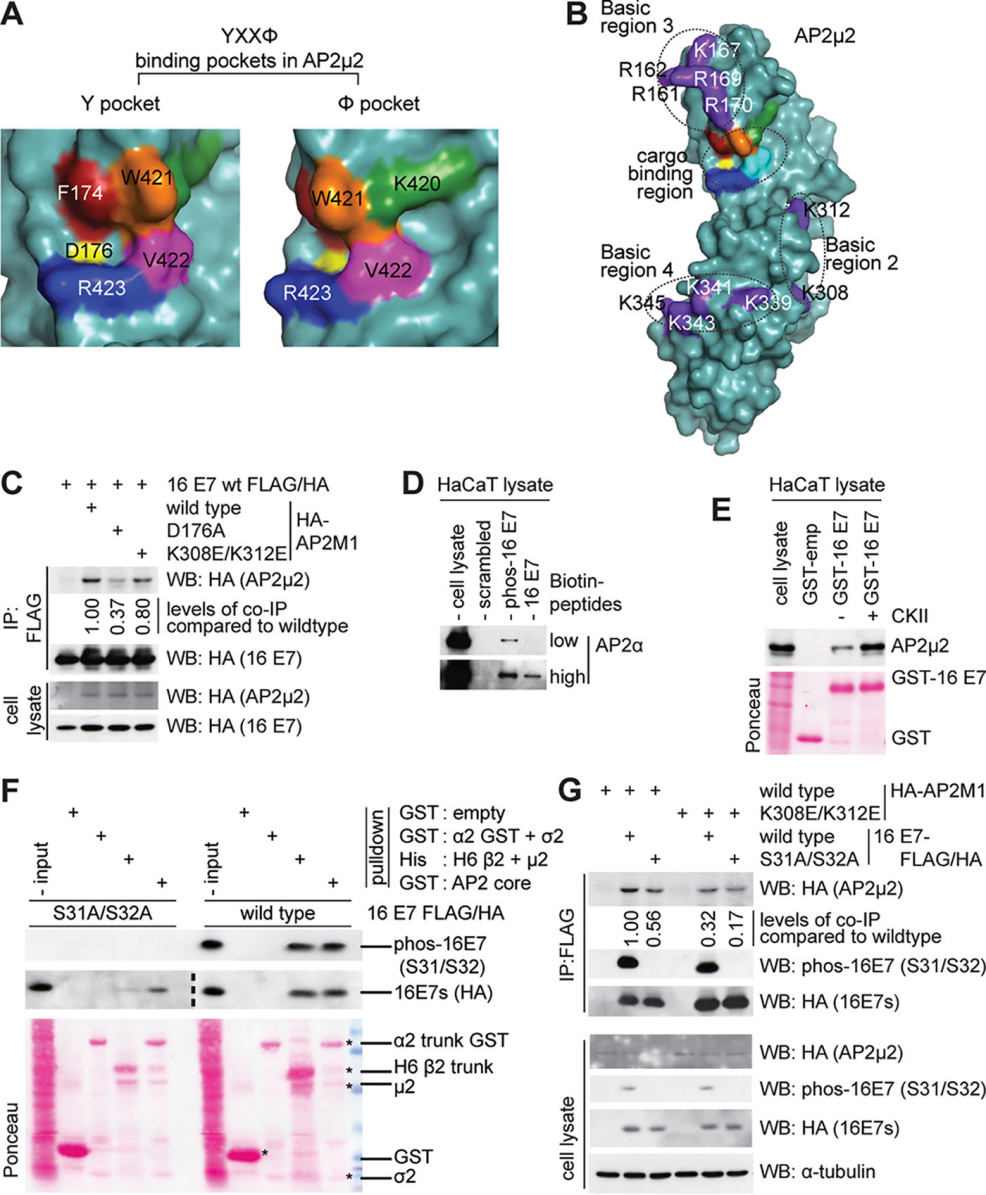
Hydrophobic pockets and basic residues in AP2-μ2 are important for interaction with HPV-16 E7 in a CKII phosphorylation-dependent manner. (A) Surface representation showing residues forming a binding site for the YxxØ motif in AP2-μ2 (PDB code 2VGL) (32) generated using PyMOL. (B) Surface representation showing basic regions in AP2-μ2 as described in references 20, 58, and 59 (PDB code 1I31) (60) generated using PyMOL. (C) Coimmunoprecipitation using anti-FLAG agarose beads and lysates of HEK 293 cells overexpressing HA-AP2M1 wild-type or D176A or K308E/K312E and C-terminally FLAG/HA-tagged HPV-16 wild type, to analyze HPV-16 E7 residues important in AP2M1 interaction. Immune complexes were analyzed by SDS-PAGE and Western blotting. (D) Peptide pulldown assay using biotinylated peptides corresponding to the HPV-16 E7 N29S CR2 region with phosphorylated or nonphosphorylated serine residues, using HaCaT cell extract. Purified streptavidin-biotin peptide complexes were analyzed by SDS-PAGE and Western blotting to analyze levels of bound AP2-α. (E) CKII phosphorylation of HPV-16 E7 enhances interaction with AP2-μ2, shown using pulldown assay with purified GST-empty, GST-16 E7, and GST-phospho-16 E7 and HaCaT cell lysates. Bound AP2-μ2 was analyzed using SDS-PAGE and Western blotting using mouse anti-AP2-μ2 antibody, and levels of GST proteins are shown by Ponceau staining. (F) Pulldown assay. GST-empty or AP2 hemi-complexes or AP2 core proteins bound to GST or Ni-agarose were incubated with HEK 293 cell lysates overexpressing C-terminally FLAG/HA-tagged HPV-16 E7 wild type or the S31A S32A mutant. Bound E7 and levels of E7 phosphorylation were analyzed by SDS-PAGE and Western blotting. Levels of GST-empty and AP2 complex subunits are shown by Ponceau staining of nitrocellulose membrane. (G) Coimmunoprecipitation using anti-FLAG agarose beads in HEK 293 cell lysates overexpressing HA-AP2M1 wild type or the K308E K312E mutant and C-terminally FLAG/HA-tagged HPV-16 wild type or the S31A S32A mutant to analyze whether the CKII phosphorylation site (acidic residues) or phosphorylation of E7 and basic residues in AP2M1 contribute to the interaction between AP2M1 and HPV-16 E7. Immune complexes were analyzed by SDS-PAGE and Western blotting. Levels of HPV-16 E7 phosphorylation were detected using phospho-HPV-16 E7 antibody (S31/S32).

It has been previously shown that Yxxφ motif-containing cargoes interact with the hydrophobic pocket residues F174, D176, W421, V422, and R423 of the AP2-μ2 subunit (19) (Fig. 4A and B). We therefore asked whether this hydrophobic pocket in AP2-μ2 is responsible for interacting with HPV-16 E7. Indeed, in a coimmunoprecipitation assay using HEK 293 cells overexpressing either HA-AP2M1 wild-type or the D176A mutant, together with HPV-16 E7, we saw that mutation in the tyrosine binding pocket (D176A) in AP2-μ2 also perturbs the interaction with HPV-16 E7 (Fig. 4C).

Furthermore, basic residues in AP2-μ2 (Fig. 4B) have also been shown to interact with the phosphoserine acidic cluster motif (PSAC) (20), and there is an acidic patch just downstream of the LXCXE motif in the E7 CR2 (Fig. 2A), where the serine residues of several high- and low-risk E7s are phosphorylated by casein kinase II, albeit with differing kinetics (33–35). This is similar to the acidic patch found in HIV-1 Nef protein that is important for interaction via the dileucine-based motif (36, 37).

To investigate whether such an acidic patch could contribute to binding the AP2-μ2 subunit, we performed a preliminary biotinylated peptide pulldown using a phospho-peptide corresponding to the CR2 region of the HPV-16 E7 N29S variant. We found that this phospho-peptide pulled down more AP2α subunit from HaCaT cell extract than the nonphosphorylated peptide (Fig. 4D). Furthermore, in pulldown assays using purified GST fusion proteins of HPV-16 E7 and phospho-HPV-16 E7 incubated with HaCaT cell lysate, we saw an enhanced interaction of AP2-μ2 with phospho-HPV-16 E7 compared with non-phospho-HPV-16 E7 (Fig. 4E). We extended these analyses using purified AP2 hemicomplexes or AP2 core, incubating them with HEK 293 cell extracts overexpressed with HPV-16 E7 wild type or S31A/S32A mutant, and then performing either GST or Ni-nitrilotriacetic acid (Ni-NTA) agarose pulldowns. As seen in Fig. 4F, the nonphosphorylable S31A/S32A mutant of HPV-16 E7 weakly associates with AP2 core or AP2-β2-μ2 hemicomplex, while phosphorylable HPV-16 E7 wild type binds strongly to both AP2 core and AP2-β2-μ2 hemicomplex. It is also noteworthy that E7’s association with AP2-α2-σ2 hemicomplex is undetectable, compared with its association with AP2-β2-μ2 hemicomplex, which suggests that the μ2 subunit is chiefly responsible for this interaction, with the acidic cluster contributing through an avidity effect.

To analyze the role of the basic residues of AP2-μ2 in this interaction *in vivo*, we used one of the several basic patch mutants in AP2-μ2, K308E K312E (20), and performed immunoprecipitation assays as before. In Fig. 4C and G we show that the double mutant K308E K312E also disrupts the association with HPV-16 E7, albeit to a lesser degree. Furthermore, when the HPV-16 E7 CKII phosphorylation site mutant (S31A S32A) and the AP2-μ2 basic patch mutant (K308E K312E) are overexpressed in HEK293 cells, co-immunoprecipitation assays show that the interaction between AP2-μ2 K308E K312E and HPV-16 E7 S31A S32A is compromised, compared with the interaction between the wild-type counterparts (Fig. 4G). This suggests that, in addition to a bona fide Yxxφ motif, phospho-serine residues in the acidic patch are important for E7’s interaction with basic residues in AP2-μ2, and in cells this might be chiefly modulated by CKII phosphorylation of E7.

### Is interaction of HPV-16 E7 with AP2 complex important for BRK cell transformation?

**FIG 5:**
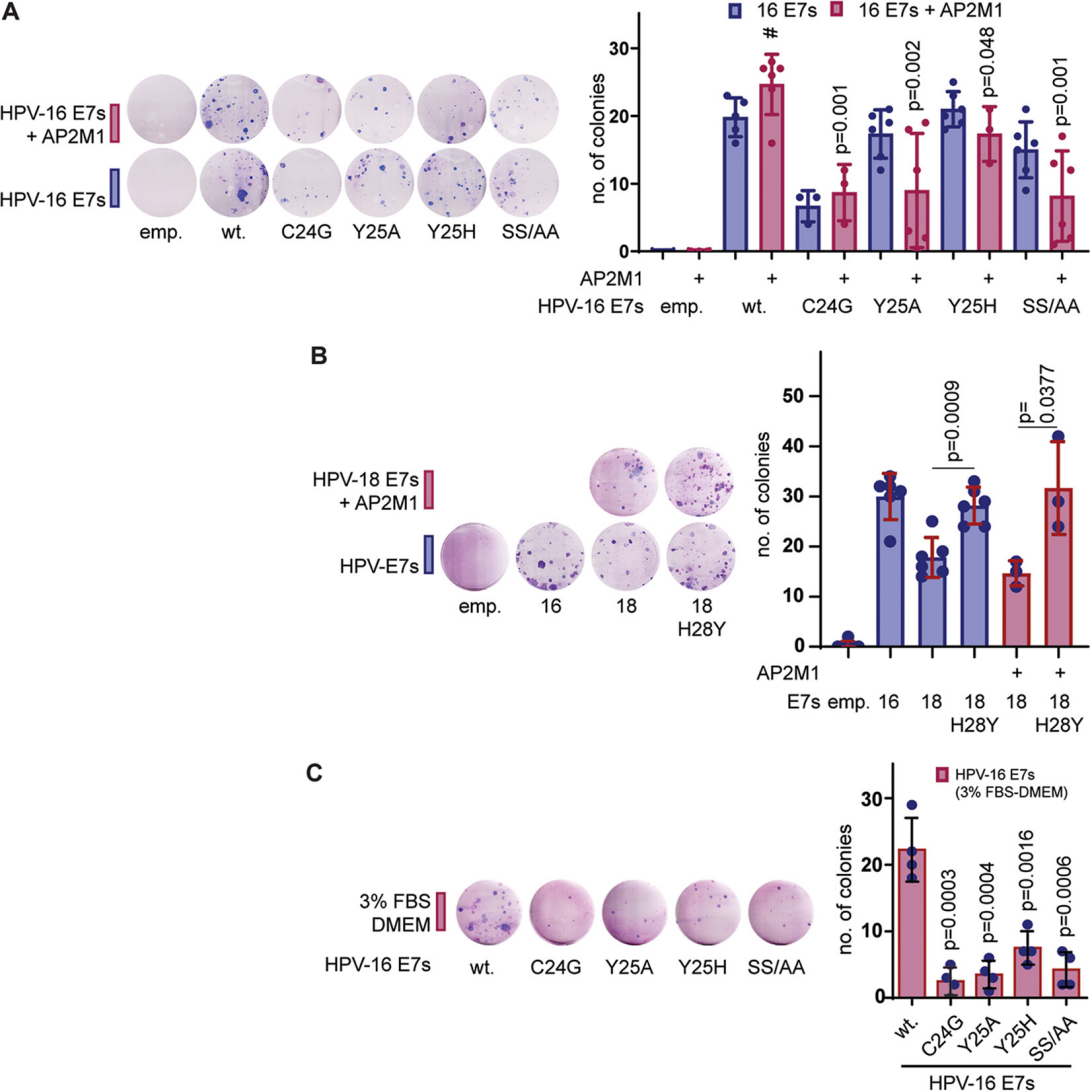
Residues in HPV-16 E7 responsible for binding the AP2 complex are important for BRK cell transformation. (A) Representative Giemsa-stained plates showing transformed BRK cell colonies after transfection of primary BRK cells with activated Ras and pJ4Ω HPV-16 E7 wild type or mutants, as indicated alone or in combination with AP2M1, and selected using G418 over 2 weeks. Mean numbers of colonies are shown in the histogram. The *P* values of Student’s *t* test, comparing the mean number of colonies obtained from cotransformation of HPV-16 E7 wild type and AP2M1 (indicated with #), with the numbers from cotransformation of HPV-16 E7 mutants and AP2M1, are indicated above their respective bars. (B) Representative Giemsa-stained plates showing transformed BRK cell colonies after transfection of primary BRK cells with activated Ras and pJ4Ω HPV-16 E7 or -18 E7 wild type or HPV-18 E7 H28Y mutant alone or in combination with AP2M1 and selected using G418 over 2 weeks. The *P* values of Student’s *t* test between the mean number of colonies with HPV-18 E7 and HPV-18 E7 H28Y alone and in cotransformation with AP2M1 are indicated. (C) Representative Giemsa-stained plates showing transformed BRK cell colonies after transfection of primary BRK cells with activated Ras and pJ4Ω HPV-16 E7 wild type or mutants, as indicated, and selected using G418 over 2 weeks in low-serum medium (3% fetal bovine serum [FBS]-DMEM). Mean numbers of colonies are shown in the histogram. The *P* values of Student’s *t* test, comparing the mean numbers of colonies obtained from HPV-16 E7 wild type and mutants, are indicated above their respective bars.

The overlap between E7’s pocket-protein interaction region and residues responsible for binding the AP2 complex was intriguing and led us to ask if the AP2-HPV-16 E7 interaction has any role in cellular transformation. To examine this, we performed baby rat kidney (BRK) cell transformation assays, as described previously (27). As shown in Fig. 5A, the pRB binding-defective mutant, HPV-16 E7 C24G, has a dramatically reduced ability to transform cells compared with the wild-type HPV-16 E7, as described before (38, 39). However, AP2-μ2 binding-defective mutants (Y25A, Y25H, and CKII mutants) showed similar transforming ability as the wild-type E7 (blue bars), although when AP2-μ2 was coexpressed, wild-type HPV-16 E7 gave rise to more transformed colonies (difference not significant at 95% confidence interval [CI]) than HPV-16 E7 alone; and when we used AP2-μ2 binding-defective mutants of E7 (Y25A, Y25H, or CKII mutant), coexpression of AP2-μ2 largely abolished the ability of these mutants to bring about cell transformation (pink bars), suggesting that HPV-16 E7’s interaction with AP2 can potentially augment its transforming ability in BRK cells.

In addition, we see a significant increase in the number of transformed colonies induced by the H28Y mutant compared with wild-type HPV-18 E7 (Fig. 5B) alone or coexpressed with AP2-μ2, emphasizing the importance of E7’s Yxxφ motif and its interaction with AP2-μ2 in cell transformation. These results indicate that, for optimal transforming activity, E7 must target the AP2 complex and that increased levels of AP2 can actually enhance E7’s transforming activity. However, where E7 is unable to target AP2, overexpression has a strong tumor-suppressive potential.

HPV-16 E7 is a major transforming protein and shows transforming potential even under low-growth factor conditions (28). To further delineate the role of this motif in cell transformation, we tested the transforming ability of the mutant HPV-E7s under conditions of reduced-serum (Dulbecco’s modified Eagle’s medium [DMEM] with 3% fetal bovine serum). As shown in Fig. 5C, the wild-type HPV-16 E7 can very efficiently transform BRK cells as before; however, the AP2-μ2-binding-defective mutants (Y25A, Y25H, or CKII mutant) have significantly reduced ability to transform these cells, confirming that there is a critical requirement for this motif, and potentially for interacting with AP2-μ2, for efficient transformation by HPV-16 E7, particularly under low-nutrient conditions.

### Does interaction of HPV-16 E7 with AP2-μ2 affect endocytic cargoes?

**FIG 6:**
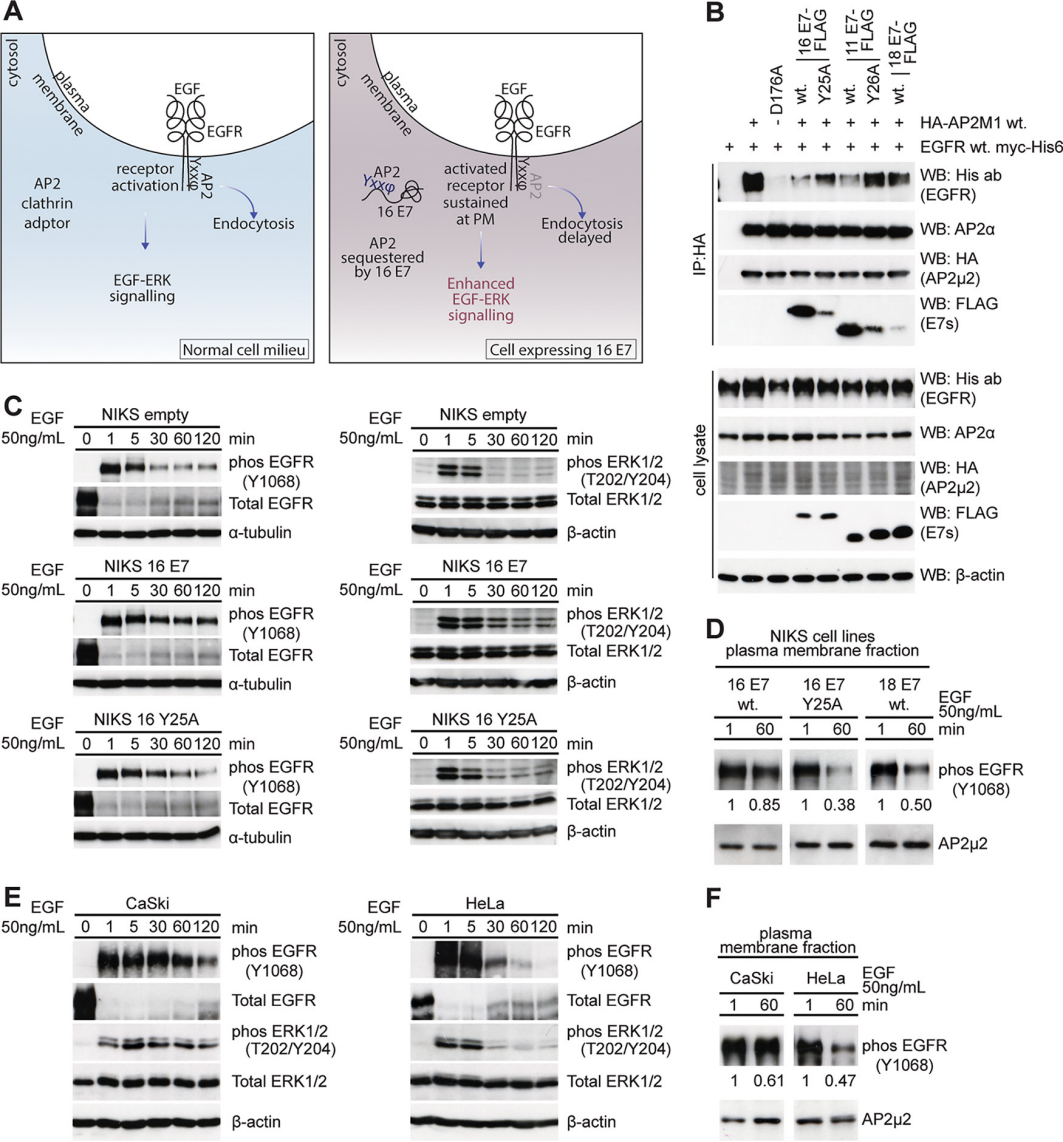
EGFR signaling is sustained in cells expressing E7 with the AP2 binding motif. (A) Cartoon showing normal cells and cells expressing HPV-16 E7 upon EGF stimulation. Normal cells activate EGFR, relaying the signal downstream to the ERK pathway, and finally, leading to growth and proliferation. Activated EGFR is endocytosed through the AP2 complex, leading to degradation or recycling of the receptor. In cells expressing HPV-16 E7, however, endocytosis is probably delayed due to sequestration of AP2 by E7 occupying the cargo binding motif, allowing sustained EGFR-ERK signaling. (B) Perturbation of AP2 interaction with EGFR in wild-type and mutant E7-expressing cells shown by coimmunoprecipitation assay. Cell lysates from HEK 293 cells cotransfected with myc-His6-tagged EGFR WT, HA-AP2-μ2 WT or D176A mutant, HPV-16 E7 WT or Y25A mutant, HPV-11 E7 WT or Y26A mutant, or HPV-18 E7 WT as indicated, were used to coimmunoprecipitate EGFR and E7s using anti-HA agarose beads to concentrate AP2-μ2. Immune complexes were analyzed by SDS-PAGE and Western blotting for bound EGFR, and E7s, and AP2 subunits as shown. (C) Levels of phospho-EGFR [Y1068] and phospho-Erk1/2 [T202 Y204] in NIKS cell lines stably expressing empty vector, HPV-16 E7 WT, or HPV-16 Y25A starved overnight and then treated with 50 ng/mL EGF over 0, 1, 5, 30, 60, and 120 min. (D) Levels of phospho-EGFR (Y1068) in the plasma membrane fraction of cells stably expressing HPV-16 E7 WT or HPV-16 E7 Y25A or HPV-18 E7 after 1 and 60 min upon 50 ng/mL EGF stimulation of cells starved overnight. (E) Levels of phospho-EGFR (Y1068) and phospho-Erk1/2 (T202 Y204) in CaSki and HeLa cells over 0, 1, 5, 30, 60, and 120 min upon 50 ng/mL EGF stimulation of cells starved overnight. (F) Levels of phospho-EGFR (Y1068) in plasma membrane fraction of CaSki and HeLa cells after 1 and 60 min upon 50 ng/mL EGF stimulation of cells starved overnight.

The intriguing results from the interaction and BRK transformation assays led us to wonder whether E7 might affect the endocytic cargoes trafficked into endosomes by interacting with the AP2 endocytic complex and potentially affecting signaling receptors, such as tyrosine kinase receptors (Fig. 6A). To study this, we chose the epidermal growth factor receptor (EGFR), which interacts with AP2-μ2 via the EGFR tyrosine residue 974 (19, 40), and this is important for its internalization upon activation (41, 42). To analyze whether expression of E7 could perturb the AP2-μ2-EGFR interaction, we performed coimmunoprecipitation analyses of HEK 293 cells overexpressing myc-His-tagged EGFR wild-type (WT) and HA-AP2-μ2 WT, together with either FLAG-tagged HPV-16 E7 WT or Y25A mutant. We also included HPV-11 E7 wild type and Y26A mutant and HPV-18 E7 wild type in this assay. HA-AP2-μ2 was precipitated using anti-HA-agarose beads, and coprecipitated proteins were analyzed by SDS-PAGE and Western blotting. As seen in Fig. 6B, we could reproduce the results of previous studies where EGFR clearly precipitates with wild-type AP2-μ2 but not with a D176A mutant of the AP2-μ2 tyrosine binding motif. Strikingly, coimmunoprecipitation of EGFR with wild-type AP2-μ2 is abrogated in cells expressing wild-type HPV-16 E7 but is regained in cells expressing the HPV-16 E7 Y25A mutant, suggesting that E7 may occupy a cargo binding motif in AP2-μ2, possibly hindering the endocytic internalization of receptor proteins such as EGFR. We also saw similar perturbation of the AP2-μ2/EGFR interaction in cells expressing wild-type HPV-11 E7, which is rescued in cells expressing the 11 E7 Y26A mutants defective in binding to AP2-μ2. In cells expressing HPV-18 E7, defective in binding with AP2-μ2, the interaction with EGFR is preserved. Taken together, these results suggest that HPV-E7s with the tyrosine motif competitively occupy the cargo-binding motif of AP2, potentially modulating EGFR endocytosis.

AP2-dependent internalization of EGFR is one of the main means of regulating EGFR endocytosis (43). Given that HPV-16 E7 would competitively occupy the cargo binding motif of AP2-μ2, it would potentially delay EGFR internalization, leading to sustained activation of EGFR upon EGF stimulation, which also suggests that HPV-16 E7-expressing cells would have higher total levels of EGFR on the cell surface than wild-type cells (Fig. 6A). To analyze this, we used stable NIKS cell lines expressing either empty vector, HPV-16 E7 wild-type, or the HPV-16 E7 Y25A mutant. These NIKS cell lines were then analyzed for time-dependent EGFR activation (phosphorylation of Y1068) upon EGF stimulation after overnight serum starvation, using SDS-PAGE and Western blotting of whole-cell lysates. As seen in Fig. 6C, all three NIKS cell lines showed profound activation of EGFR (phospho-Y1068) as soon as 1 min after EGF treatment; however, this activation was only sustained in NIKS lines expressing wild-type HPV-16 E7. Furthermore, phospho-extracellular signal-regulated kinase 1/2 (ERK1/2), a downstream target of EGFR activation, follows the same sustained activation pattern as EGFR activation (Fig. 6C). We also analyzed the levels of phospho-EGFR in the membrane fractions of the NIKS stable lines at 1 and 60 min post-EGF treatment after overnight starvation (Fig. 6D) and saw that phospho-EGFR is more highly retained in NIKS-16 E7 wild type than in NIKS-16 E7 Y25A. We also saw a similar downregulation of surface EGFR in the membrane fraction of HPV-18 E7 NIKS stable cells upon EGF chase between 1 and 60 min, suggesting that activated EGFR is sustained in the membrane in E7-expressing cells only where E7 sequestrates the AP2-μ2 cargo binding motif, potentially delaying clathrin-AP2-dependent EGFR endocytosis.

We also examined the EGFR activation upon EGF treatment in cervical tumor-derived cell lines CaSki and HeLa, as before. The results in Fig. 6E and F, show that HPV-16 E7-expressing CaSki cells sustain EGFR activation upon EGF treatment, leading to the concomitant activation of phospho-ERK1/2, whereas HPV-18 E7-expressing HeLa cells cannot sustain EGFR-ERK signaling, and the activation signal disappears within 30 min of EGF treatment. Furthermore, the levels of phospho-EGFR in membrane fractions of CaSki cells are higher after 1 h, compared with after 1 min of EGF treatment, while in similarly treated HeLa cells, there are lower levels of phospho-EGFR in the membrane fraction after 1 h compared with 1 min, suggesting that competitive binding of E7 to AP2-μ2 leads to sustained EGFR activation and enhanced EGFR-ERK signaling in HPV-16-positive CaSki cells, while this does not occur in HeLa cells, further highlighting important differences in the mechanisms of action of HPV-16 and HPV-18 E7 oncoproteins.

## DISCUSSION

In this study we have identified a novel biochemical activity, common to the E7 oncoproteins from many HPV types and residing within the LXCXE pRb binding domain, that confers association with components of the AP2 complex and thus affects the regulation of clathrin-mediated endocytic cargo transport. Biologically, this appears to play a major role in the ability of E7 to bring about cell transformation and, in the absence of such an activity of E7, the AP2 complex can act as a potent tumor suppressor.

Previous proteomic studies had indicated the potential for E7 to interact with components of the clathrin endocytic transport complex (14). Further analysis of the HPV-16 E7 sequence identified a perfect consensus for AP2-μ2 recognition between residues 25-YEQL-28, upstream of the acidic patch that defines the CKII recognition motif. Such AP2-μ2 recognition motifs have also been reported to be important for AP2-μ2 recognition in a number of cargoes, including furin (21, 44, 45). This potential AP2-μ2 recognition sequence is present in E7 proteins from both high- and low-risk HPV types, suggesting that it is not uniquely linked with E7 transforming activity but reflects a major conserved function, spanning multiple HPV types. Intriguingly, the AP2-μ2 consensus site is partly embedded within the LXCXE pRb recognition motif, again highlighting the incredible functional diversity within these small DNA tumor virus oncoproteins.

Using purified components *in vitro*, we first showed that HPV-16 E7 can interact directly with AP2-μ2 and, using mutational analysis, we finely mapped the residues within E7 and AP2-μ2 required for this association. This analysis also allowed us to precisely separate E7’s pRb binding activity from its ability to interact with AP2-μ2. Intriguingly, HPV-18 E7 lacks the consensus recognition sequence for AP2-μ2, but mutation of a single amino acid at residue 28 makes HPV-18 E7 look like HPV-16 E7, and the ability to interact with AP2-μ2 is conferred. Most importantly, we were able to show, in cells derived from cervical cancers, that HPV-16 E7 can interact strongly with the AP2 complex, indicating that the interaction does, indeed, occur *in vivo* and, furthermore, that phosphorylation of E7 by CKII enhances its association with AP2-μ2, as has been observed for other classical cargoes of the AP2 complex (21, 44, 45). This raises the important question of how E7 might target pRb and AP2M1 within the same cell. However, E7 is not solely a nuclear protein, and we favor the hypothesis that the LXCXE motif confers pRb association within the nuclear pool, while the cytoplasmic pool of E7 targets the AP2 complex through the overlapping 25-YEQL-28 motif.

At present we have no evidence to suggest that E7 modulates the level of AP2-μ2 protein expression; rather, it would appear that E7 simply occupies the cargo-interacting motif of the complex, potentially competing for cargo recognition during endocytosis. Indeed, we present compelling evidence that in the case of EGFR, E7 contributes to maintaining EGFR signaling by delaying its recognition by the AP2 complex, thereby maintaining its surface expression and growth-promoting signaling. A major future goal will be to more fully understand the landscape of cellular proteins whose clathrin-AP2-dependent transport is modulated by the HPV E7 oncoproteins.

In the current article we show that the interaction of high-risk E7 with AP2 contributes to transforming ability potentially through EGFR signaling. However, the E7/AP2 interaction is also conserved among many low-risk E7 proteins, indicating that support of transforming activity in high-risk types is subordinate to a more general activity linked to the successful completion of the viral life cycle. Indeed, enhancement of proliferative signaling through EGFR may be one such mechanism, but further studies are required to gain a fuller understanding of the global effects of E7 upon AP2-mediated cargo transport. It is also interesting to note that the E7/AP2 interaction is enhanced by CKII phosphorylation of E7, where high-risk types are substantially better substrates than the low-risk types (35), which also most likely contributes to differences between the different viruses with respect to the biological consequences of the AP2 interaction.

As noted above, the AP2-μ2 recognition motif is found in both high- and low-risk E7 oncoproteins; hence, it is not unique to transforming HPV types. However, AP2-μ2 recognition appears to have an important role to play in the ability of HPV-16 E7 to induce cell transformation. Clearly, loss of AP2-μ2 binding results in a modest reduction of cell transforming activity, albeit not to the same extent as loss of pRb recognition; however, restoration of AP2-μ2 recognition in HPV-18 E7 clearly augments its ability to bring about cell transformation. However, when transformation assays were performed under reduced growth factor conditions (3% serum medium), the ability to transform cells is severely compromised for E7 mutants that cannot interact with AP2-μ2, while wild-type E7 can transform cells almost as well as under regular growth factor conditions. This result again highlights the potential importance of this interaction in conferring enhanced proliferative potential under conditions of low nutrients. This also reflects nicely the previous studies also showing the that CKII phosphorylation of E7 plays an important role in maintaining proliferation in low-nutrient conditions (28), and it is tempting to speculate that the activities are connected through CKII regulation of interaction with both pRB and AP2, subsequently maintaining growth factor signaling such as that shown here for EGFR.

Taken together, these results define a new and unexpected mechanism by which E7 can modulate clathrin-mediated endocytosis and cargo selection.

## MATERIALS AND METHODS

### Cells.

The HEK 293, HaCaT, CaSki, and SiHa cell lines were obtained from the American Type Culture Collection (ATCC) and maintained in Dulbecco’s modified Eagle’s medium (DMEM) supplemented with 10% fetal bovine serum, glutamine (300 μg/mL), and penicillin-streptomycin (100 U/mL). The normal immortal keratinocytes (NIKS) cells (46, 47) were kindly provided by John Doorbar and were grown in F medium (3:1 [vol/vol] F12:DMEM medium) supplemented with 5% fetal calf serum, 0.4 μg/mL hydrocortisone, 5 μg/mL insulin, 8.4 ng/mL cholera toxin, 10 ng/mL EGF, 24 ng/mL adenine, and 100 U/mL penicillin/streptomycin as described previously (48).

### Plasmid constructs, cloning, and transfections.

The following plasmids were purchased from Addgene: μ2-HA-WT (Addgene plasmid no. 32752) and μ2-HA-D176A (Addgene plasmid no. 32754). The μ2-HA-K308E/K312E mutant was generated using a modification of the QuikChange site-directed mutagenesis system (Stratagene) according to the manufacturer’s instructions with the following primers: K308E/K312E forward primer 5′-GTC AAG GTG GTC ATC GAG TCC AAC TTC GAG CCC TCA CTT CTG-3′ and K308E/K312E reverse primer 5′-CAG AAG TGA GGG CTC GAA GTT GGA CTC GAT GAC CAC CTT GAC-3′. The GST-tagged pGEX-2T HPV-16 E7 wild type has been previously described (49). The pET-23a(+)-HPV16E7-RGS6xHis plasmid (50) and the C-terminal FLAG/HA-tagged pCMV HPV-16 E7 plasmid (51) were kind gifts from Karl Münger. The C-terminal FLAG/HA-tagged pCMV HPV-16 E7 S31A/S32A has been previously described (28). The C-terminal FLAG/HA-tagged pCMV HPV-16 E7 mutants C24G, Y25A, Y25H, L28A, S31E S32E, and LXH (Y23L Y25H) were generated using the following primers: C24G forward primer 5′-ACA ACT GAT CTC TAC GGT TAT GAG CAA TTA AAT-3′ and C24G reverse primer 5′-ATT TAA TTG CTC ATA ACC GTA GAG ATC AGT TGT-3′, Y25A forward primer 5′-CTG ATC TCT ACT GTG CTG AGC AAT TAA ATG A-3′ and Y25A reverse primer 5′-TCA TTT AAT TGC TCA GCA CAG TAG AGA TCA G-3′, Y25H forward primer 5′-ACT GAT CTC TAC TGT CAT GAG CAA TTA AAT GAC-3′ and Y25H reverse primer 5′-GTC ATT TAA TTG CTC ATG ACA GTA GAG ATC AGT-3′, L28A forward primer 5′-TCT CTA CTG TTA TGA GCA AGC AAA TGA CAG CTC-3′ and L28A reverse primer 5′-GAG CTG TCA TTT GCT TGC TCA TAA CAG TAG AGA-3′, S31E S32E forward primer 5′-GAG CAA TTA AAT GAC GAG GAA GAG GAG GAG GAT GA-3′ and S31E S32E reverse primer 5′-TCA TCC TCC TCC TCT TCC TCG TCA TTT AAT TGC TC-3′, and LXH (Y23L Y25H) forward primer 5′-GAC AAC TGA TCT CCT CTG TCA TGA GCA ATT AAA TG-3′ and LXH (Y23L Y25H) reverse primer 5′-CAT TTA ATT GCT CAT GAC AGA GGA GAT CAG TTG TC-3′. The FLAG-tagged pCMV HPV-16 E7 was generated by inserting a stop codon between FLAG and HA in pCMV C-terminal FLAG/HA-tagged HPV-16 E7 by *in vitro* mutagenesis as described before using the following primers: forward primer 5′-GAT GAC GAT GAC AAG TAA GAT GGA GGA TAC-3′ and reverse primer 5′-GTA TCC TCC ATC TTA CTT GTC ATC GTC ATC-3′.

The C-terminal FLAG/HA-tagged pCMV HPV-18 E7, HPV-11 E7, and HPV-31 E7 plasmids were kindly provided by Anita Szalmas. The C-terminal FLAG/HA-tagged pCMV HPV-18 E7 H28Y was generated by mutagenesis using H28Y forward primer 5′-GTT GAC CTT CTA TGT TAC GAG CAA TTA AGC GAC-3′ and H28Y reverse primer 5′-GTC GCT TAA TTG CTC GTA ACA TAG AAG GTC AAC-3′. The C-terminal FLAG-tagged HPV-11 (52) and -18 E7s (28) have been previously described. The C-terminal FLAG-tagged HPV-11 E7 Y26A mutant was generated by site-directed mutagenesis as described earlier using forward primer 5′-GTA GGG TTA CAT TGC GCT GAG CAA TTA GAA GAC-3′ and reverse primer 5′-GTC TTC TAA TTG CTC AGC GCA ATG TAA CCC TAC-3′.

The two bi-cistronic vectors pMW H6β2-trunk + μ2-myc and pMW α-trunk GST + σ2 (32) were kindly provided by David Owen. The HA-tagged pRb expression construct was kindly provided by James DeCaprio. The pJ4Ω 16E7 plasmid and EJ-ras expression plasmids have been described previously (53). The pJ4Ω 16E7 mutants, Y25A, Y25H, L28A, LXH, and S31A S32A, were generated by site-directed mutagenesis using primers as described above.

pPB-HPV-16 E7 WT FLAG/HA, pPB-HPV-16 E7 Y25A FLAG/HA, pPB-HPV-18 E7 FLAG/HA, and pPB-HPV-11 E7 FLAG/HA were generated by restriction digestion of pCMV HPV-16 E7 WT FLAG/HA, pCMV HPV-16 E7 FLAG/HA Y25A, pCMV HPV-18 E7 FLAG/HA, and pCMV HPV-11 E7 FLAG/HA at BamHI and EcoRI sites to extract E7 open reading frames (ORFs) and were inserted into the same restriction sites in pPB-MSC empty vector. pCMV-HyPBase and pPB-MSC empty vector were kindly provided by Guido Papa (54).

All constructs were sequence-verified using Sanger sequencing.

HEK293 cells were transfected using the calcium phosphate precipitation method (55).

### Production and purification of recombinant proteins.

Purified AP2 complex/core was produced by cotransforming two bi-cistronic vectors, pMW H6β2-trunk + μ2-myc (ampicillin resistance) and pMW α-trunk GST + σ2 (kanamycin resistance), in Escherichia coli (BL21 DE3 pLysS) on LB plates containing ampicillin and kanamycin. Single colonies were then grown in 40 mL LB broth with ampicillin and kanamycin at 37°C overnight. The expression was induced adding IPTG (isopropyl-β-d-thiogalactopyranoside) to a final concentration of 0.2 mM and lowering the temperature to 22°C for 14 h, and cells were then harvested by centrifugation. The bacterial pellet was resuspended in phosphate-buffered saline (PBS)/1% Triton X-100 and sonicated on ice twice for 30 s with 30 s between each pulse. The lysate was centrifuged at 10,000 rpm for 15 min, and supernatant was transferred to a fresh tube. Then glutathione agarose was added to the supernatant with the recommended concentration of protease inhibitors (Calbiochem protease inhibitor cocktail 1) and incubated on a rotating wheel at 4°C for 1 h. The agarose beads were then washed 3 times with PBS/1% Triton and finally resuspended in 1 mL PBS/1% Triton plus 1mL glycerol and stored at −80°C until use.

Hemicomplexes of H6β2-trunk + μ2-myc and α-trunk GST + σ2 were prepared as described above using Ni^2+^-nitrilotriacetic acid (NTA) agarose and glutathione agarose beads, respectively.

For production of purified C-terminally His_6_-tagged HPV-16 E7, the pET-23a(+)-RGS6xHis bacterial expression plasmid was transfected into E. coli (BL21 DE3 pLysS) on LB plates containing ampicillin. An isolated bacterial clone harboring the plasmid was grown in LB culture medium, as described above, and induced using 0.2 mM IPTG overnight at 30°C. The cells were then harvested by centrifugation and resuspended in Tris buffer (20 mM Tris, pH 8, 300 mM NaCl, 10 mM imidazole, 0.5% NP-40) and sonicated to obtain a clear lysate, as described above. C-terminally His_6_-tagged HPV-16 E7 was then column-purified from the lysate using Ni^2+^-nitrilotriacetic acid (NTA) agarose columns from Qiagen (QIAexpress system) according to the manufacturer’s instructions. Purified protein was aliquoted and stored at −80°C until use.

Production and purification of GST-empty and GST-16 E7 has been described previously (52). For production of GST-phosho-16 E7, E. coli BL21 DE3 cells were coexpressed with GEX-2T HPV-16 E7 wild-type and pCDF CKII duet vector.

pCDF CKII duet vector expressing α and β subunits of CKII was kind gift from Yong Xiong and John Guatelli.

### *In vitro* translation and glutathione-agarose pulldown.

The μ2-HA-WT plasmid was translated *in vitro* using the Promega TNT kit and radiolabeled with [^35^S] methionine (Perkin Elmer) according to the manufacturer’s instructions. The purified GST-fusion proteins were then incubated with *in vitro*-translated proteins for 1 h at 4°C. Protein complexes were then washed with 1× PBS containing 1% Triton X-100 and were analyzed by SDS-PAGE and autoradiography.

### Antibodies, Western blotting, and coimmunoprecipitation.

The following antibodies were used for Western blotting: mouse monoclonal anti-α-tubulin, mouse monoclonal anti-HA-peroxidase (clone HA-7), mouse monoclonal anti-FLAG-M2-peroxidase from Sigma, 6×-His Tag antibody (MA1-21315) from Thermo Fisher, mouse monoclonal anti-HPV-18 E7 antibody (F-7), mouse monoclonal anti-HPV-16 E7 antibody (NM2), mouse monoclonal anti-GFP antibody (B-2), mouse monoclonal EGFR antibody (A-10) (sc-373746), mouse monoclonal β-adaptin (A-5) (sc-74423), mouse monoclonal anti-ERK1/2 antibody (C-9) (sc-514302) from Santa Cruz Biotechnology, mouse monoclonal anti-Rb (G3-245), purified mouse anti-adaptin α (610502), purified mouse anti-AP50 (611350) from BD Pharmingen, rabbit anti-phospho-EGF receptor (Tyr1068) (D7A5) (monoclonal antibody [MAb] no. 3777), rabbit anti-phospho-p44/42 mitogen-activated protein kinase (MAPK) (Erk1/2) (Thr202/Tyr204) (D13.14.4E) (MAb no. 4370), rabbit monoclonal anti-Rb antibody (D20) (MAb no. 9313) from Cell Signaling, goat polyclonal anti-HA tag antibody (ab215069), and rabbit monoclonal anti-AP2M1 antibody (EP2695Y) from Abcam. HPV-16 E7 pS31/S32 peptide antibodies have been described before (28). Secondary anti-rabbit horseradish peroxidase (HRP), anti-goat HRP, and anti-mouse HRP antibodies were obtained from Dako.

For Western blotting, total cell extracts were obtained by lysing the cells directly in 2× SDS-PAGE sample buffer and were then separated by SDS-PAGE and blotted on a 0.22-μm nitrocellulose membrane. Membranes were blocked in 5% nonfat dry milk in TBST (20 mM Tris-HCl, pH 7.5, 150 mM NaCl, 0.1% Tween 20) for 1 h and probed with the appropriate primary and secondary antibodies. The blots were then developed using the ECL Western blotting detection reagent (GE Healthcare) according to the manufacturer’s instructions.

For coimmunoprecipitation, cells were harvested using lysis buffer (10 mM Tris, pH 7.4, 100 mM NaCl, 1 mM EDTA, 1 mM EGTA, 1% Triton X-100, 10% glycerol, 0.1% SDS, 0.5% deoxycholate, 1 mM NaF, protease inhibitor cocktail I [Calbiochem]) and centrifuged at 13,000 rpm for 15 min. Cell lysates for overexpressed proteins were incubated with FLAG- or HA-agarose beads as indicated for 4 h at 4°C followed by washing times with the lysis buffer. For coimmunoprecipitation using endogenous proteins, cell lysates were incubated with 1 to 2 μg of the indicated antibody overnight at 4°C. After incubation, immune complexes were incubated further with protein A or G beads (immobilized protein A 300 [Repligen] or protein G Sepharose [Amersham Biosciences]) for 90 min at 4°C, followed by four washes in the NP-40 buffer (150 mM NaCl, 50 mM HEPES, pH 7.4, 0.1% NP-40). Immunoprecipitates were then run on SDS-PAGE and analyzed by Western blotting.

### Preparation of HaCaT cell lysate and peptide pulldown.

Soluble proteins were extracted from 80% confluent HaCaT cells by incubation for 10 min on ice in lysis buffer (50 mM HEPES, pH 7.4, 150 mM NaCl, 1 mM MgCl_2_, 1% Triton X-100, protease inhibitor cocktail I [Calbiochem]). The cell lysate was collected by scraping the plate into a prechilled Eppendorf tube followed by centrifugation at 14,000 rpm in a benchtop centrifuge for 10 min at 4°C.

Next, 500 μg of each peptide (non-phospho-peptide: biotin-LQPETTDLYCYEQLSDSSEEEDEIDG; phospho-peptide: biotin-LQPETTDLYCYEQLpSDpSpSEEEDEIDG; scrambled peptide: biotin-RRLQRTVEQR) in lysis buffer, was bound to streptavidin-conjugated magnetic Sepharose beads (Streptavidin-MagSepharose, GE Healthcare) by incubation at 4°C on a rotating wheel for 1 h and then washed three times with lysis buffer. The cell extract was precleared by incubation with empty streptavidin-conjugated magnetic beads at 4°C on a rotating wheel for 1 h. After removal of the preclearing beads, the extract was incubated at 4°C on a rotating wheel for a further 2 h with each of the biotinylated peptides bound to streptavidin-conjugated magnetic Sepharose beads. The beads were washed three times with lysis buffer without protease inhibitors, transferred to fresh Eppendorf tubes, and washed twice more with lysis buffer without protease inhibitors or Triton X-100. Equal amounts of beads were then analyzed using SDS-PAGE and Western blot analysis (56).

### Primary baby rat kidney (BRK) cell transformation assay.

Primary epithelial cells were obtained from the kidneys of 9-day-old Wistar rats as described previously (53). After 24 h, the cells were transfected by calcium phosphate precipitation (55) with plasmids expressing EJ-ras or HPV-16 E7 or with plasmids expressing μ2-HA. The cells were maintained under G418 selection for 2 weeks and then fixed and stained with Giemsa. For transformation assays in reduced growth factor conditions, DMEM supplemented with 3% fetal bovine serum was used. The stained colonies were counted, and a *t* test was used to compare the mean numbers of colonies formed in different sets of transfections, as described in “Statistical Analysis.”

### Generation of NIKS stable cell lines.

NIKS empty, NIKS 16 E7 WT, and NIKS 16 E7 Y25A were generated using *piggyBac* technology (57). Briefly, NIKS cells were transfected with the pCMV-HyPBase (57) and transposon plasmids pPB-MCS empty, pPB-HPV-16 E7 or pPB-HPV-16 E7 Y25A, and pPB-HPV-18 E7 or pPB-HPV-11 E7 using a ratio of 1:2.5 with FuGENE HD transfection reagent (Promega) according to the manufacturer’s instructions.

The cells were maintained in NIKS medium as described above for 3 days, and then the cells were incubated with same medium with 1.25 μg/mL puromycin (Sigma-Aldrich) for 4 days to allow the selection of cells expressing the gene of interest.

### Preparation of plasma membrane fraction from cells.

Plasma membrane fractions from the cells were prepared with the Minute endosome isolation and cell fractionation kit from Invent Biotechnologies (catalog [cat.] no. ED-028) using the manufacturer’s instructions. Briefly, NIKS stable lines, CaSki, and HeLa cells were starved overnight with serum-free medium and then treated with 50 ng/mL of EGF for 1 min and 60 min. Cells (10 × 10^6^ to 30 × 10^6^) were collected using Trypsin-EDTA and low-speed centrifugation (600 × *g* for 5 min). The cells were washed once with ice-cold PBS and resuspended in 500 μL of buffer A to incubate in ice for 7 min. After incubation, the cell suspension was vigorously vortexed for 15 s and immediately transferred to filter cartridge and spun for 16,000 × *g* for 30 s. The filter cartridge was discarded, and the pellet was then vigorously vortexed for 10 s and centrifuged at 700 × *g* for 2 min. The supernatant was transferred to a fresh 1.5-mL microcentrifuge tube and centrifuged at 4°C for 60 min. After centrifugation, the supernatant was transferred to a fresh 1.5-mL tube, and the pellet (plasma membrane fraction) was dissolved in Minute denaturing protein solubilization reagent (cat. no. WA-009; Invent Biotechnologies). This membrane fraction then was mixed with 2× sample buffer for SDS page and Western blotting.

### Statistical analysis.

All experiments were performed at least thrice, and data are shown as the mean and standard error of the mean. Statistical significance was calculated using GraphPad Prism software. For the quantification of protein levels from Western blots, the films were scanned, and the intensity of bands was measured using ImageJ software. The final relative quantification values are the ratio of net band to net loading control. To compare two groups, the paired Student’s *t* test was performed. A *P* value below 0.05 was considered statistically significant, and a *P*-value above 0.05 was considered nonsignificant.
